# Companion Dogs in Vietnam: Exploring Characteristics of Owned and Ideal Dogs

**DOI:** 10.3390/ani16040574

**Published:** 2026-02-12

**Authors:** Jemma Sheppard, Joanna Shnookal, Dac L. Mai, Huy N. Vo, Phillipa D. Bandis, Pauleen C. Bennett, Deanna L. Tepper

**Affiliations:** School of Psychology and Public Health, La Trobe University, Bendigo, VIC 3552, Australia; 21214171@students.latrobe.edu.au (J.S.); j.shnookal@latrobe.edu.au (J.S.); jimmy.mai@latrobe.edu.au (D.L.M.); n.vo@latrobe.edu.au (H.N.V.); 19352078@students.latrobe.edu.au (P.D.B.)

**Keywords:** preferred traits, companion animal, human–animal interaction, anthrozoology, behaviour

## Abstract

As the first domesticated species, dogs have been shaped by human needs, values, and social structures, resulting in culturally specific expectations of their behaviour. While much of the existing research on companion animal preferences (referred to as ideal traits) has focused on Western contexts, rapidly increasing companion dog populations in Eastern countries mean that preferences in diverse cultures are important to investigate. Considering owner satisfaction is influenced by the match between dogs’ traits and owners’ culturally specific ideals, understanding ideal dog traits may help minimise welfare concerns, including relinquishment and maltreatment of companion dogs. A sample of 312 Vietnamese adults (M age = 27.31, SD = 6.65) were recruited via convenience sampling through Facebook and Instagram. Participants completed translated versions of the 44-item Ideal Dog Scale. Exploratory factor analysis identified four dimensions of ideal dog traits in Vietnam: ‘Calmness’, ‘Energy/Capability’, ‘Affection/Health’, and ‘Cleanliness’. This differs from Australia, where the ideal dog is ‘calm/compliant’, ‘sociable/healthy’, ‘energetic/faithful/protective’, ‘socially acceptable’, and ‘non-aggressive’, and from Italy, where the ideal dog is ‘calm’, ‘sociable and healthy’, ‘well trained and adaptable’, ‘energetic’, and ‘easy to manage‘. For participants with a current dog, most owned a non-desexed, mixed breed male dog. This study deepens psychological insight into how socioecological contexts may shape perceptions of ideal companion animals. It also allows Vietnamese individuals to understand traits that would lead to the best dog–owner ‘match’, improving dog owner wellbeing and dog welfare.

## Simple Summary

Most research on ‘ideal’ companion dogs, referring to dogs with traits that align with what owners prefer for the purpose of social support or emotional connection, has focused on Western countries, despite growing dog ownership in Eastern contexts. Vietnamese participants completed a 44-item questionnaire, the Ideal Dog Scale, comprising demographic questions and questions about their ideal dog, and for current dog owners, their actual dog’s traits. Four ‘ideal’ traits were identified: Calmness, Energy/Capability, Affection/Health, and Cleanliness. This is in comparison to Australia and Italy, where the ideal dog is typically viewed as calm, sociable and energetic. For Vietnamese participants with a current dog, dogs were typically male, not desexed, a mixed breed, and small or medium sized. The findings highlight how cultural and environmental factors shape perceptions of companion animals. Accordingly, the right match between owners and dogs with ideal traits, shaped by cultural context, may enhance owner satisfaction. In turn, understanding owner preferences can aid in minimising the relinquishment and maltreatment of companion dogs.

## 1. Introduction

As the first domesticated animal, dogs share a long history with humans, during which they developed unique characteristics [1,2]. More recently, dogs were selectively bred for functional purposes such as guarding, hunting and herding livestock [2,3]. This selective breeding led to behavioural specialisations and physiological differences resulting in distinct breeds [4,5,6]. Although many cultures used dogs for similar roles, different breeds often emerged independently across countries, suggesting environmental and cultural factors may have influenced the development of specific dog traits [7,8,9].

In contemporary society, the primary role of dogs has shifted toward companionship [10], influencing the wellbeing of owners and their pets [11,12]. For example, dog owners have lower risk factors associated with cardiovascular disease, depression, and stress [13,14,15,16]. Additionally, dogs facilitate social interactions, particularly with strangers [17]. Therefore, dog owners potentially benefit from greater physical and mental wellbeing, and community connectivity. However, owners of dogs which exhibit behavioural issues, such as aggression, may experience heightened stress, sadness, and interpersonal conflicts [18]. This may contribute to social isolation, loneliness and decreased attachment to the dog [18,19]. Consequently, dog behavioural problems increase the chances of relinquishment [20], leading to welfare concerns, including elevated stress for the animals involved [12]. As such, finding the right dog–owner ‘match’ is crucial for fostering fulfilling relationships with companion dogs.

Identifying a dog whose traits align with owners’ preferences helps determine the right dog–owner ‘match’ [21,22]. Generally, the ideal companion dog in Australia scores highly on five behavioural factors: ‘calm/compliant’, ‘sociable/healthy’, ‘energetic/faithful/protective’, ‘socially acceptable’, and ‘non-aggressive’ [3]. In an Italian study, owners described their ideal dog as ‘calm’, ‘sociable and healthy’, ‘well trained and adaptable’, ‘energetic’, and ‘easy to manage’ [23]. While Australian and Italian owners value calmness, sociability, energy, and health, the Italian study also emphasised trainability, adaptability, and ease of management. These findings indicate that cultural factors may influence owner perceptions of the ideal dog’s behavioural characteristics, which may contribute towards positive welfare outcomes for companion dogs.

Appearance-related characteristics also shape perceptions of the ideal dog. In Australia, characteristics such as sex, purebred status, and coat texture are generally unimportant, albeit with some preferences for medium-sized, short-haired, and low-shedding dogs [3,24]. Similarly, the Italian study indicates that coat texture, length and colour, sex, and purebred status are generally unimportant [23]. Therefore, behavioural traits may be prioritised over appearance when individuals identify their ideal companion dog. However, in the Italian study, participants’ ideal and actual dogs differed in reproductive status, size, coat colour, breed type, and age of acquisition [23]. Overall, this suggests that expectations for the ideal dog are often unmet.

Culture is likely an important determinant of human preferences for dog traits. While human–dog companion relationships are widespread in many Eastern societies, studies exploring human-canine bonds have been largely shaped by Western perspectives [25]. Furthermore, the popularity of dogs as pets in Vietnam has substantially increased since the early 2000s [26], presenting a unique opportunity to investigate the role of culture in shaping human–dog relationships. Additionally, historical influences have played a significant role in shaping cultural differences among Vietnam’s regions. In the North, Chinese colonisation displaced indigenous hunter–gatherer groups and strongly influenced lifestyle and social structures [26,27]. Meanwhile, central Vietnam was shaped by the multi-ethnic Champa Kingdom, and through external influences, particularly from India [27,28,29]. As such, Eastern cultural forces historically influenced both northern and central Vietnam. In contrast, southern Vietnam has experienced greater exposure to Western culture through French colonisation and American military operations during the Vietnam War [30,31], potentially leading to a stronger adoption of Westernised cultural values.

These historical differences help explain regional variations in modern-day human–dog relationships across Vietnam. For instance, in northern Vietnam, people more commonly approve of dogs for human consumption, a practice rooted in historical Chinese dietary customs [26]. While the human–dog relationship in the Champa Kingdom is not well documented, it is known that in many Eastern cultures, including India, dogs were traditionally kept for guarding purposes [26,32,33]. This may explain the common use of guard dogs in central Vietnam today [26]. In the South, while no data currently exists on dog ownership, Western influences may have encouraged a greater value placed on companion-based relationships with dogs. Therefore, contemporary ideals surrounding companion dog traits may vary regionally, shaped by these historical and cultural differences.

Vietnam continues to undergo significant economic growth and urbanisation, widening cultural differences between rural and urban areas [34], which may influence perceptions of the ideal dog. For example, Vietnam’s urban economic growth has contributed to higher incomes in cities compared to rural regions [35,36], higher educational attainment [37], apartment living [38,39], changes to traditional gender roles [34,40], and greater exposure to Western social media, particularly among Vietnamese youth [41,42]. In other countries, such factors have led individuals to keep smaller dogs for companionship rather than utility [43,44]. Comparatively, rural areas of Vietnam typically have larger properties for agriculture and are more likely to retain traditional values and customs, therefore being more suited to keeping larger dogs valued for herding or guarding [45,46,47,48].

As establishing a strong dog–owner match is essential for fostering close relationships and supporting owner wellbeing [11,21] and dog welfare [12], it is important to understand people’s preferences for ideal dog traits. Since little research has examined ideal dog traits in Eastern societies, including Vietnam, this study aimed to identify key traits constituting the ‘ideal’ companion dog in Vietnamese society. As this is the first study to investigate these perceptions in a Vietnamese context, no specific a priori hypotheses or directional predictions were tested. Instead, analyses were exploratory and guided by existing literature on regional, socioeconomic, and cultural variation in Vietnam. This study also explored characteristics of participants’ actual dogs.

## 2. Materials and Methods

This project was approved by the La Trobe University Human Research Ethics Committee (Approval Number: HEC25197). The project consisted of two stages: a pilot study to test the accuracy and cultural appropriateness of the Vietnamese translation of the Ideal Dog Scale [3,24], followed by a larger study exploring the traits of actual and ideal dogs. A copy of the English version of the questionnaire is available in the Supplementary Materials.

### 2.1. Phase 1: Pilot Study

The Ideal Dog Scale [3,24] has not been previously translated into Vietnamese. For this phase, 15 Vietnamese participants aged between 21 and 38 years (M = 27.33, SD = 4.72; 13.3% female, 80% male) were recruited using the research team’s personal networks on Facebook and Instagram, two of Vietnam’s most popular social media platforms [49]. This sample size was appropriate per Sousa and Rojjanastrirat’s [50] recommendations. Inclusion criteria included being a current Vietnamese resident, fluent in Vietnamese, and 18 years or older.

The standard translation and back-translation approach was applied to all items to maintain the scale’s psychometric integrity while developing items for Vietnamese participants [51,52]. This translation was conducted in the Northern dialect following Vietnam’s national standard [53] by two members of the research team (DLM and HNV), who are native Vietnamese speakers with expertise in research design and questionnaire translation. Each item was reviewed for cultural and contextual relevance, with minor adjustments applied to ensure appropriateness for the Vietnamese context. For example, ‘travels calmly and quietly in the car’ was adapted to include ‘motorcycle or other vehicles’ to better reflect common modes of transportation in Vietnam [54], with the intention of capturing tolerance for transport-related contexts rather than implying equivalence of sensory or physical experience.

The pilot study’s purpose was to reveal any issues with item comprehension or translation inaccuracies, improving the reliability and validity of the items before implementation in the main study. Participants were asked if each proposed item was ‘clear’ or ‘unclear’. If participants thought an item was unclear, they were asked ‘how to revise?’.

Pilot study results were reviewed, and adjustments made based on participant feedback to enhance the clarity of the translation. For example, both the original phrase ‘Thích được ôm ấp và vuốt ve’ and the revised phrase ‘Thích được âu yếm và ôm ấp’ translate into ‘enjoys being cuddled and hugged’, but pilot participants suggested the latter reflects affectionate and tender nuances implied in the English phrasing. In cases where the original translation more accurately represented the original English item, it was retained despite participant suggestions for modification.

### 2.2. Phase 2: Main Study

#### 2.2.1. Participants

In the main phase of this study, participants were recruited through a snowballing technique using the research team’s personal networks on Facebook and Instagram. The inclusion criteria were identical to the pilot phase, and included being a current Vietnamese resident, fluent in Vietnamese, and 18 years or older. A total of 433 participants commenced the study.

#### 2.2.2. Demographics

The first section of the survey asked about participant demographics (e.g., year of birth, gender, region of Vietnam the participant resides in). Participants were also asked whether they currently own any dogs.

#### 2.2.3. Ideal Dog Scale

The Ideal Dog Scale was initially developed by King et al. [3] to describe characteristics important to Australian dog owners, with this scale recently updated by Power et al. [24]. Section B of Power et al.’s [24] adapted version of the Ideal Dog Scale was used in the present study to measure the value people place on behavioural dog traits. The measure contains 44 questions measured on 5-point Likert scales, ranging from 1 (extremely unimportant) to 5 (extremely important). An example item is, ‘learns new tasks quickly’. A modified version of section F was used to ask participants about characteristics of their current dog, comprising 22 items.

Statistical measurement of the Ideal Dog Scale’s reliability and validity has not been reported. However, results from King et al.’s [3] and Power et al.’s [24] Australian studies were similar, indicating that it has test–retest reliability. Furthermore, Diverio et al. [23] successfully used the scale in an Italian sample, a culturally and linguistically distinct setting from the original Australian studies, indicating cross-cultural reliability.

#### 2.2.4. Procedure

The study was hosted on REDCap, an online data application for managing surveys and databases. The full survey took 15–20 min to complete. Data were collected in September 2025, with the survey open for 1.5 weeks. No individual reimbursement was provided, but participation was incentivised through offering a donation of 17,000 VND per survey completion to Laws for Paws, a not-for-profit Vietnamese animal charity based in Ho Chi Minh City.

#### 2.2.5. Data Analysis

Raw survey data were imported from REDCap into Microsoft Excel for manual cleaning [55]. Participants were removed from the data set if the full survey was incomplete (*n* = 121). The data were then imported into the Statistical Package for Social Sciences (SPSS; version 30.0.0.0) programme for analysis. Descriptive analyses were conducted on the demographic information provided by the participants.

Inspection of skewness and kurtosis indicated mild to moderate deviations from normality across several items. The Ideal Dog Scale was therefore condensed using Exploratory Factor Analysis (EFA) using principal axis factoring with oblimin rotation. The Kaiser–Meyer–Olkin measure of sampling adequacy was above 0.6, and Bartlett’s Test of Sphericity was statistically significant. Scree plots and a Monte Carlo simulation were reviewed to identity the number of factors to extract. Items in each factor were then averaged to generate subscale scores.

Group differences across categorical demographic and contextual variables were examined using multivariate analysis of variance (MANOVA), with the four ideal-dog subscales entered as simultaneous dependent variables. Several categorical predictors were collapsed prior to analysis to ensure adequate cell sizes and stable multivariate estimates (e.g., current region, current urbanicity, property size, and education attainment). Due to the deviation from normality and correlated nature of the subscales, Pillai’s Trace was used as the primary multivariate test statistic. Where multivariate effects were significant, follow-up univariate analyses of variance (ANOVAs) were conducted for each subscale. To control for Type I error across multiple tests, Bonferroni-adjusted alpha levels (0.0125) were applied.

Associations with continuous and ordinal predictors (age and perceived wealth) were examined using Spearman’s rank-order correlations, with the same Bonferroni adjustments applied.

## 3. Results

### 3.1. Sample Demographics

Participant demographics for the main study are summarised in Table 1. Ages ranged from 18 to 52 years (M = 27.31, SD = 6.65). Of the 312 final participants, 71.2% were female (n = 222) and 26.6% (n = 83) were male. Most participants lived in South Vietnam (n = 246, 78.8%), followed by North Vietnam (n = 43, 13.8%) and the middle of Vietnam (n = 22, 7.1%). Most participants currently owned a dog (n = 171, 54.8%), and almost all participants had previous experience owning a dog (n = 282, 90.4%).

### 3.2. Ideal Dog: Physical Characteristics

Most participants did not have a strong preferred sex for their ideal dog (n = 214, 68.6%), followed by a preference for male dogs (n = 76, 24.4%). De-sexed or neutered dogs were preferred by over half of the participants (n = 168, 53.8%), while under half did not consider the sexual status of their ideal dog to be important (n = 125, 40.1%). Most participants did not consider breed type important (n = 213, 68.3%), followed by a preference for purebred dogs (n = 58, 18.6%).

Small (4–10 kg; n = 113, 36.2%) and medium (10–20 kg; n = 105, 33.7%) sized dogs were considered ideal by most participants, while several participants did not have a strong preference for size (n = 54, 17.3%). Half of participants preferred low-shedding (n = 156, 50.0%), followed by no-shedding (n = 66, 21.2%) dogs. For fur texture and length, participants could select multiple choices; participants mostly preferred medium fur (n = 149, 47.8%), followed by short fur (n = 115, 36.9%), or had no specific preference (n = 94, 30.1%). For texture, over half of participants preferred a smooth coat (n = 181, 58.0%), with others having no strong preference (n = 116, 37.2%). Coat colour was similarly not important for most participants (n = 175, 56.1%), followed by a smaller preference for brown coloured fur (n = 53, 17.0%).

### 3.3. Ideal Dog: Behavioural Characteristics

An initial exploratory factor analysis (EFA) found 11 components with eigenvalues above 1, with a cumulative percentage of variance accounted for of 64.31%. An inspection of the scree plot suggested four or five factors would be appropriate, while simulations from the Monte Carlo PCA for Parallel Analysis programme indicated five factors would be suitable.

A five-factor solution was tested first, suppressing coefficients with absolute values below 0.4 [56]. Many items failed to load adequately or demonstrated cross-loadings across successive iterations, indicating an unstable solution. In contrast, a four-factor solution demonstrated stronger and more interpretable loadings, and therefore was retained as the most meaningful structure, as seen in Table 2.

From the results of the EFA, four behavioural factors were created, ‘Calmness’ (Cronbach’s α = 0.87; M = 3.78; SD = 0.58), ‘Energy/Capability’ (Cronbach’s α = 0.88; M = 3.17; SD = 0.65), ‘Affection/Health’ (Cronbach’s α = 0.81; M = 3.82; SD = 0.68), and ‘Cleanliness’ (Cronbach’s α = 0.84; M = 4.33; SD = 0.71). All mean subscale scores were above the mid-point, with particularly strong support for Cleanliness. The lowest mean score was for Energy/Capability.

### 3.4. Associations Between Demographics and Ideal Dog Behavioural Characteristics

#### 3.4.1. Gender

A multivariate analysis of variance (MANOVA) comparing the four ideal-dog subscales across participant gender revealed no significant effect; Pillai’s Trace = 0.007, F(4, 300) = 0.55, *p* = 0.697, partial η^2^ = 0.007.

#### 3.4.2. Age

Participant age was not significantly correlated with ‘Calmness’ (ρ = 0.06, *p* = 0.314), ‘Energy/Capability’ (ρ = 0.02, *p* = 0.802), or ‘Cleanliness’ (ρ = −0.04, *p* = 0.518). Age showed a small negative correlation with ‘Affection/Health’ at the nominal level (ρ = −0.14, *p* = 0.017), but this association was not significant after the Bonferroni adjustment.

#### 3.4.3. Education

There were no significant group differences across education, Pillai’s Trace = 0.034, F(8, 614) = 1.33, *p* = 0.225, partial η^2^ = 0.017.

#### 3.4.4. Wealth

Wealth was not significantly correlated with ‘Calmness’ (ρ = 0.07, *p* = 0.20), ‘Energy/Capability’ (ρ = 0.07, *p* = 0.21), ‘Affection/Health’ (ρ = 0.11, *p* = 0.05), or ‘Cleanliness’ (ρ = 0.02, *p* = 0.69).

#### 3.4.5. Region

There were no significant group differences across region for where the participant currently resides (North/Middle Vietnam versus South Vietnam), Pillai’s Trace = 0.008, F(4, 306) = 0.58, *p* = 0.676, partial η^2^ = 0.008.

#### 3.4.6. Urbanicity

A MANOVA revealed a significant effect for urbanicity, referring to whether participants currently reside in urban versus non-urban areas of Vietnam, for the four ideal-dog scales; Pillai’s Trace = 0.049, F(4, 307) = 3.917, *p* < 0.05, partial η^2^ = 0.049. Although the multivariate effect of urbanicity on the four ideal-dog subscales was statistically significant, none of the follow-up one-way ANOVAs were significant, as shown in Table 3.

#### 3.4.7. Property Size

A MANOVA revealed a significant effect for property size, Pillai’s Trace = 0.034, F(12, 915) = 1.816, *p* < 0.05, partial η^2^ = 0.023. However, follow-up one-way ANOVAs did not reveal any significant differences between property sizes for the four ideal dog traits after the Bonferroni correction (α = 0.0125), as shown in Table 4.

### 3.5. Actual Dog Characteristics

While 171 participants reported currently owning one or more dogs, only dog owners who completed the second section of the survey were included in this section (n = 122). Actual dog characteristics are described in Table 5. Most participants owned male dogs (n = 75, 61.5%). A majority of the dogs owned were not neutered or desexed (n = 67, 54.9%). Most dogs were mixed breed (n = 67, 54.9%), followed by purebred dogs (n = 50, 41.0%), then designer dogs (n = 5, 4.1%).

Small dogs were the most common (4–10 kg; n = 60, 49.2%), followed by medium-sized dogs (10–20 kg; n = 49, 40.2%). Most participants owned dogs that shed moderately (n = 50, 41.0%) or were low-shedding (n = 45, 36.9%), followed by those that shed heavily (n = 14, 11.5%) and those that did not shed (n = 13, 10.7%). Most dogs had medium-length fur (n = 65, 53.3%). The dog’s fur texture was mostly described as smooth (n = 81, 66.4%). Dogs mostly had a brown coat (n = 42, 34.4%), followed by black (n = 22, 18.0%), white (n = 21, 17.2%), multi-coloured (n = 19, 15.6%), and other colours (n = 18, 14.8%).

Of the participants who owned a dog, many were satisfied with their dog’s behaviour (n = 58, 47.5%) and health (n = 70, 75.4%), while many were very satisfied with their physical appearance (n = 63, 51.6%). The majority of owners named their dogs (n = 116, 95.1%), and both Vietnamese and English names were used.

## 4. Discussion

To expand the predominantly Western-centric understanding of human–animal companion relationships, this study aimed to examine the ‘ideal’ companion dog in Vietnam. This exploration highlights culturally specific ideal traits that exemplify the ideal dog–owner match and may contribute to enhanced owner satisfaction and, consequently, reduced companion dog relinquishment and maltreatment. Four traits were identified: ‘Calmness’ (M = 3.78, SD = 0.58), ‘Energy/Capability’ (M = 3.17, SD = 0.65), ‘Affection/Health’ (M = 3.82, SD = 0.68), and ‘Cleanliness’ (M = 4.33, SD = 0.71). While the factors represent latent dimensions, that is, underlying trait constructs inferred from patterns of responses rather than directly observed preferences, the mean scores across these four dimensions allow interpretation and insight into which traits were evaluated more positively. Vietnamese participants tended to evaluate dogs more favourably on Calmness, Affection/Health, and Cleanliness, with desired levels of Energy/Capability showing a lower mean and somewhat greater variability.

Several of these traits closely mirror Australian preferences, indicating that there are similarities in ideal dog traits across Eastern and Western countries. For example, Australian, Italian and Vietnamese participants generally value dogs that show calmness in routine situations and energy during play or exercise (e.g., walks calmly on a leash) [23,24]. However, in our current study, evaluations of the ideal dog’s energy and capability were more mixed in Vietnam. Notably, items loading most strongly on this factor reflected traits such as hunting capability, possibly reflecting a broader preference towards physical drive and alertness, even among urban participants. That this differs from Australia may reflect differences in lifestyle, living arrangements, or access to outdoor space, as well as differences in how energy-related traits are conceptualised across contexts. Both Australian and Vietnamese participants tended to evaluate affectionate behaviours positively (e.g., enjoys being cuddled and hugged). However, the current findings uniquely highlight the importance of ‘Cleanliness’ as an ideal trait in Vietnam, possibly reflecting practical concerns tied to the prevalence of free-roaming dogs and disease transmission in Vietnam [57].

Across both Australia and Italy, respondents tended not to prioritise the sex, type of dog (e.g., purebred) or coat characteristics of their ideal dog, indicating that these features were broadly unimportant for most participants [23,24]. For size, Italian participants preferred large dogs, while medium dogs were the preference in Australia [23]. In comparison to Italy, Australian respondents showed a strong preference for neutered dogs [24]. The ideal dog in Vietnam seemed to more closely resemble the Australian ideal dog, with desexed or neutered dogs preferred by over half the respondents, and small to medium dogs preferred over larger dogs. Sex, breed type, and coat colour were not typically viewed as important, but short to medium fur and a smooth coat were preferred by participants. From a welfare perspective, preferences for small to medium body size, smooth coats, and short to medium fur likely reduce the time and resources required for routine care, potentially lowering owner burden and frustration. When care demands align with owner capacity and expectations, dogs may be more likely to experience consistent and appropriate care [21].

Relevant information is sparse, but ideal companion dog traits may differ across Eastern cultures. Research in Japan identified calmness, sociability, boldness, and trainability as key personality traits in Japanese dog breeds [58]. While there is consistency between Japanese dogs being bred to be calm, and the current study identifying ‘Calmness’ as a preferred trait in Vietnam, several notable differences were present. Traits such as Japanese dogs being bold and easily trained were not reflected as ideal traits in our Vietnamese sample, whereas ‘Cleanliness’ was important in Vietnam. Once again, this may reflect that non-scavenging behaviours may be more important in Vietnam than in countries such as Japan due to higher rabies risk, more variable vaccination coverage, and more frequent unregulated outdoor roaming [57,59]. While it is not yet possible to directly compare ideal traits across Eastern countries, it is expected that ideal traits differ across Asia, and this suggests that regulation and public health conditions may be important in shaping the ideal dog within countries.

On an exploratory basis, this study also explored relationships between the four identified dog traits and demographic factors. Historical cultural influences have contributed towards distinct regional identities within Vietnam [27,28,29,30,31], while higher income, greater educational attainment, and greater exposure to Western media have widened rural–urban cultural differences [34]. Despite this, there were no significant differences in the mean scores for the four factors between the different regions of Vietnam, or for age, gender, educational attainment, or wealth. In comparison, urbanicity and property size demonstrated small but significant multivariate effects, although no individual dog traits differed significantly across these variables following correction for multiple testing. This suggests that ideals about dogs may be largely culturally shared across Vietnam, rather than being constrained by environmental context or differences in living space. As discussed below, however, sampling bias may also have contributed to these findings.

Although many of the Vietnamese participants reported a preference for desexed dogs, data from current owners indicated that over half of participants owned an intact dog. While this discrepancy has not yet been empirically examined, it likely reflects limited veterinarian access, which has previously been highlighted as a concern in Vietnam [60,61]. Additionally, we also note that two participants were unable to report their current dog’s sex or reproductive status (*n* = 2, 1.6% each). This may reflect ownership of free-roaming or loosely supervised dogs, where contact and veterinary involvement may be limited, making such information less visible or less relevant to owners. For the animals, the mismatch between preferred and actual practices in desexing can have welfare implications. At an individual level, limited access to desexing may affect the management of reproductive behaviours, which can influence roaming, aggression, and owner–dog conflict, potentially increasing stress for both dogs and owners [19,62]. At the population-level, lower desexing rates may contribute to unplanned breeding and increasing numbers of free-roaming dogs, placing pressure on existing care systems and welfare resources [63]. Importantly, the expressed preference for desexed or neutered dogs suggests that owner attitudes may already be aligned with welfare-oriented practices. This highlights the need for more veterinary education in Vietnam, but also an opportunity for targeted interventions focused on improving access to veterinary services and community-based desexing programmes [61].

A limitation of this study was the reliance on convenience sampling through online recruitment, which introduced demographic biases. While only 34.43% of Vietnam’s population live in urban areas [64], 78.8% of participants in this study were currently urban residents. The sample also overrepresented adults aged 18 to 52, a group comprising 64.1% of the adult population [64]. We note that no specific age group was targeted beyond the inclusion criterion of adults aged 18 years and older, and recruitment was not stratified by region within Vietnam. This bias likely stems from younger, urban populations’ higher social media use, where recruitment occurred [49]. Additionally, as is predominant in other human–animal research [65], the study showed a gender bias, with 71.3% of participants being female. In comparison, approximately 50.23% of individuals in Vietnam are women [64]. These imbalances limit generalisability, particularly regarding rural dog–owner dynamics and older generations’ attitudes. However, since younger, urban residents are the primary demographic purchasing companion dogs in Vietnam [26,66], the sample offers a meaningful foundation for exploratory research into dog–owner relationships. Future research should use stratified sampling, including in-person recruitment of rural and older populations, to deepen theoretical understandings of diverse dog–owner relationships across Vietnam.

Overall, this research has helped identify traits underlying perceptions of the ideal dog for individuals in Vietnam. Future research could build on these findings by examining how population-level preferences for ideal dog traits relate to adoption decisions and dog–owner outcomes in Vietnam. Similar work in Australia and Italy has been instrumental in guiding breeder and buyer purchasing behaviour [23,24]. Additionally, the finding that ideal dog traits differ somewhat between Eastern and Western cultures is important. Since some dog breeds tend to exhibit specific personality traits and behavioural tendencies [4,67], this cultural difference means that not all breeds will naturally align with Vietnamese owners’ changing preferences. Social media may have shaped pet-keeping trends in Vietnam by promoting Western ideals and popular Western dog breeds [41,68]. Consequently, trending breeds may reflect traits valued in the West rather than in the Vietnamese context, potentially leading to mismatches and challenges in dog–owner relationships. However, this possibility was not directly examined in the present study and warrants further investigation. It will be important for Vietnam to develop and promote trends reflecting local preferences, rather than focusing on what is currently popular in Western countries. Future research would also benefit from directly examining the alignment between owners’ identified “ideal” dog characteristics and the behaviour of their current dog, to better understand whether owner satisfaction reflects perceived fit. 

Beyond Vietnam, perceptions of the ‘ideal dog’ are likely to influence animal welfare through their impact on acquisition decisions, owner expectations, and daily management practices. When preferred traits align with a dog’s behavioural and physical needs, dogs may be more likely to experience stable care, appropriate management, and sustained human–dog relationships [21]. Conversely, mismatches between owner ideals and dog characteristics have been associated with increased behavioural challenges, owner dissatisfaction, and instability in care, including relinquishment [19,20]. Importantly, this is likely not culturally specific but reflects a broader mechanism through which human expectations shape welfare outcomes for companion dogs. Understanding how ideals vary across cultures therefore contributes to welfare science by identifying where and why mismatches may occur.

While identifying ideal traits is a vital first step in understanding the right dog–owner ‘match’ in Vietnam, key factors, such as whether a dog’s actual temperament influences relationship quality, remain unexplored [22]. Future research may incorporate ethnographic methods to explore local attitudes toward companion dogs, behavioural observations or questionnaires to assess dog temperament, and repeated measures studies to track how relationships develop over time in this context and across other Eastern cultures. Such culturally grounded research will aid in extending understanding beyond Western frameworks and provide deeper insights to enhance owner satisfaction and dog–owner relationship quality within Vietnam and the broader Eastern context.

## 5. Conclusions

This study identified four characteristics underlying perceptions of the ideal Vietnamese companion dog: ‘Calmness’, ‘Energy/Capability’, ‘Affection/Health’, and ‘Cleanliness’. Although the structure of these traits is broadly similar to that reported in Western samples, the strong and unique emphasis on ‘Cleanliness’ may reflect local concerns regarding free-roaming dogs. Socio-cultural values likely shaped these preferences. Compared with Japan, the prioritisation of ‘Cleanliness’ may highlight cross-cultural variation among Eastern societies. Participants predominantly preferred a desexed dog that is small or medium in size, low-shedding, with a medium or short, smooth-textured coat. Comparatively, actual companion dogs in Vietnam were predominantly male, non-desexed, and mixed-breed or purebred. These dogs were also small or medium-sized, and moderate shedders, with medium-length, smooth-textured, brown fur. These findings advance understanding of how culture shapes companion-animal ideals and can inform efforts to promote more compatible human–dog relationships in Vietnam, helping to minimise companion dog relinquishment and maltreatment by enhancing owner satisfaction, which is largely influenced by the match with culturally specific ideal traits.

## Figures and Tables

**Table 1:** Demographic Characteristics of Participants (n = 312).

Characteristic	*n*	%
Gender		
Male	83	26.6
Female	222	71.2
Non-binary/third gender	2	0.6
Prefer not to say	4	1.3
Other	1	0.3
Region currently lived in		
North of Vietnam	43	13.8
Middle of Vietnam	22	7.1
South of Vietnam	246	78.8
Missing	1	0.3
Region lived in the most over lifetime		
North of Vietnam	47	15.1
Middle of Vietnam	50	16.0
South of Vietnam	213	68.3
Missing	2	0.6
Description of current community type		
Urban	246	78.8
Rural	29	9.3
Suburban	36	11.5
Other	1	0.3
Type of community lived in the most over lifetime		
Urban	199	63.8
Rural	72	23.1
Suburban	38	12.2
Other	2	0.6
Missing	1	0.3
Property type		
Apartment	105	33.7
House without backyard	91	29.2
House with backyard	71	22.8
Small property (less than 1000 m^2^)	26	8.3
Medium property (1000 m^2^–10,000 m^2^)	4	1.3
Other	1	0.3
Missing	2	0.6
Highest education level completed		
Higher secondary/year 12 equivalent	22	7.1
Vocational qualification	5	1.6
Advanced diploma	7	2.2
Undergraduate degree	223	71.5
Bachelor’s degree	51	16.3
Postgraduate degree (Masters, PhD)	4	1.3
Main work status		
Student	82	26.3
Employed	195	62.5
Unemployed	27	8.7
Homemaker/housewife	5	1.6
Missing	3	1.0
Perceived personal financial wealth relative to others		
Well below average	25	8.0
Below average	54	17.3
Average	149	47.8
Above average	59	18.9
Well above average	5	1.6
Prefer not to say	18	5.8
Missing	2	0.6
Main belief system		
Atheist	55	17.6
Folk Beliefs (e.g., Ancestor Worship, Mother Goddess Worship)	125	40.1
Buddhist	80	25.6
Catholic	41	13.1
Other	10	3.3
Missing	1	0.3

**Table 2:** Factor loadings and descriptive statistics from the four-factor exploratory factor analysis.

Calmness		Energy/Capability		Affection/Health		Cleanliness	
Items	Factor Loadings	Items	Factor Loadings	Items	Factor Loadings	Items	Factor Loadings
Does not growl at strangers in public areas	0.712	Has hunting capabilities	0.705	Enjoys being cuddled and hugged	0.793	Does not eat their own faeces	0.966
Does not bark at strangers in public areas	0.640	Has high energy levels	0.558	Enjoys being petted	0.787	Does not eat other animals’ faeces	0.895
Does not fight with other dogs	0.621	Enjoys large amounts of exercise	0.548	Shows affection toward me	0.712	Does not scavenge things found in the street	0.423
Does not dig inappropriately	0.596	Will bite people on command	0.536	Is constantly attentive to me	0.455		
Never jumps on people	0.581	Is protective of myself and my family	0.534	Is physically healthy	0.437		
Is not overly excitable	0.558	Learns new tasks quickly	0.495	Lives until they are at least 10 years old	0.434		
Does not escape from my property	0.556	Is confident in new surroundings	0.490				
Does not bark inappropriately	0.512	Enjoys obedience training	0.480				
Walks calmly without pulling on the leash	0.456	Does not beg for food	0.478				
Does not chase wildlife or farm animals	0.446	Likes to play rough and tumble games	0.477				
Is not destructive when left alone	0.424	Remains calm during thunderstorms or fireworks	0.444				
Behaves calmly most of the time	0.407	Barks at people who enter my property	0.409				

**Table 3:** Means, standard deviations, and one-way Analysis of Variance (ANOVA) for the effects of urban versus non-urban living on the four ideal-dog traits.

	Urban		Non-Urban		F (1, 310)	*p*	η^2^
Variable	*M*	*SD*	*M*	*SD*			
Calmness	3.79	0.55	3.76	0.65	0.20	0.652	0.001
Energy	3.14	0.62	3.27	0.75	2.29	0.132	0.007
Affection/Health	3.85	0.66	3.67	0.71	3.67	0.056	0.012
Cleanliness	4.37	0.69	4.22	0.77	2.31	0.130	0.007

**Table 4:** Means, standard deviations, and one-way Analysis of Variance (ANOVA) for the effects of property size on the four ideal-dog traits.

	Apartment		House (No Backyard)		House (Backyard)		Property (Other) ^a^		F ^b^	*p*	η^2^
Variable	*M*	*SD*	*M*	*SD*	*M*	*SD*	*M*	*SD*			
Calmness	3.79	0.55	3.76	0.53	3.88	0.58	3.76	0.55	0.94	0.420	0.009
Energy/Capability	3.14	0.62	3.13	0.63	3.36	0.67	3.19	0.69	3.51	0.016	0.033
Affection/Health	3.85	0.66	3.70	0.68	3.88	0.70	3.76	0.65	1.62	0.186	0.016
Cleanliness	4.37	0.69	4.32	0.74	4.47	0.55	4.36	0.68	1.50	0.216	0.014

Note: ^a^ = participants reported that they lived in small (less than 1000 m^2^), medium (1000 m^2^–10,000 m^2^) or large properties (more than 10,000 m^2^); ^b^ = df (3, 306).

**Table 5:** Physical Characteristics, Acquisition and Maintenance of Participants’ Currently Owned Dogs in Vietnam (n = 122).

Characteristic	*n*	%
Has a name		
No	6	4.9
Yes	116	95.1
Current age		
Under 1 year	12	9.8
Between 1 and 2 years	18	14.8
Between 2 and 8 years	66	54.1
Between 8 and 12 years	20	16.4
Over 12 years old	5	4.1
Unknown	1	0.8
Age at acquisition		
Under 1 month	37	30.3
Between 1 and 4 months	61	50.0
Between 4 and 12 months	12	9.8
Between 1 and 3 years	7	5.7
Over 3 years	1	0.8
Unknown	4	3.3
Sex		
Male	75	61.5
Female	45	36.5
Unknown	2	1.6
Desexed/castrated		
Yes	53	43.4
No	67	54.9
Unknown	2	1.6
Source of acquisition		
Pet shop	13	10.7
Breeder	8	6.6
Shelter/rescue	5	4.1
Friend/family	62	50.8
Independently bred	13	10.7
Gift	9	7.4
Found	3	2.5
Other	9	7.4
Role		
Pet/companion	89	73.0
Guard dog	26	21.3
Breeding dog	1	0.8
Other	6	4.9
Fur length		
Hairless	2	1.6
Short	34	27.9
Medium	65	53.3
Long	21	17.2
Fur texture		
Smooth	81	66.4
Wavey	20	16.4
Curly	17	13.9
Corded	4	3.3
Shedding		
No shedding	13	10.7
Low shedding	45	36.9
Moderate shedding	50	41.0
Heavy shedding	14	11.5
Coat colour		
Black	22	18.0
White	21	17.2
Brown	42	34.4
Multi-coloured	19	15.6
Other	18	14.8
Size		
Tiny (0–3 kg)	6	4.9
Small (4–10 kg)	60	49.2
Medium (10–20 kg)	49	40.2
Large (20–40 kg)	7	5.7
Breed		
Purebred	50	41.0
Mixed breed	67	54.9
Designer dog	5	4.1
Cost to maintain (weekly)		
0–150,000 VND	46	37.7
150,000–300,000 VND	57	46.7
300,000–500,000 VND	10	8.2
500,000+ VND	9	7.4
Time owner spends exercising (minutes per day)		
0	5	4.1
1–15	33	27.0
16–30	38	31.1
31–60	20	16.4
61+	26	21.3
Time owner spends grooming (minutes per week)		
0	21	17.2
1–15	38	31.1
16–30	36	29.5
31–60	19	15.6
61+	8	6.6
Dog’s primary location		
On property (inside a house and/or the yard)	98	80.3
Free roaming	20	16.4
Other	4	3.3
Dog’s primary habitat when on the owner’s property		
Inside the house	61	50.0
Inside the yard	32	26.2
Inside both the house and the yard equally	28	23.0
Other	1	0.8
Satisfaction with the dog’s behaviour		
Very dissatisfied	2	1.6
Dissatisfied	1	0.8
Neither satisfied nor dissatisfied	25	20.5
Satisfied	58	47.5
Very satisfied	36	29.5
Satisfaction with the dog’s health		
Very dissatisfied	1	0.8
Dissatisfied	5	4.1
Neither satisfied nor dissatisfied	25	20.5
Satisfied	70	57.4
Very satisfied	21	17.2
Satisfaction with the dog’s physical appearance		
Very dissatisfied	1	0.8
Dissatisfied	1	0.8
Neither satisfied nor dissatisfied	9	7.4
Satisfied	48	39.3
Very satisfied	63	51.6

Note. ‘Designer dog’ is a non-technical term commonly used to describe intentionally bred crossbreed dogs often marketed under a combined breed name (e.g., Labradoodle); ‘Purebred dog’ refers to a dog bred from parents of the same recognised breed.

## Data Availability

The data presented in this study are available on request from the corresponding author, provided that approval to release the data is obtained from the La Trobe University Human Ethics Committee.

## Supplementary Materials

The following supporting information can be downloaded at: https://www.mdpi.com/article/10.3390/ani16040574/s1, File S1: English version of survey.
