# Supplementary material for: Companion Dogs in Vietnam: Exploring Characteristics of Owned and Ideal Dogs
Source: Animals (Basel). 2026 Feb 12;16(4):574. doi: 10.3390/ani16040574 (PMC12937297; doi:10.3390/ani16040574)
Supplement: Supplementary file 1 [file animals-16-00574-s001.zip › animals-4072926-supplementary.pdf]

1. Are you 18 years of age or older?

◯ Yes
◯ No

---

2. In what year were you born?

____

---

3. Were you born in Vietnam?

◯ Yes
◯ No (In what country were you born?______)

---

4. How many years have you lived in Vietnam?

____

---

5. What area of Vietnam do you currently live in?

◯ North of Vietnam
◯ Middle of Vietnam
◯ South of Vietnam

---

6. What area of Vietnam have you spent most of your life in?

◯ North of Vietnam
◯ Middle of Vietnam
◯ South of Vietnam

---

7. In your opinion, do you currently live in a rural or urban area?

◯ Urban
◯ Rural
◯ Suburban
◯ Other (please describe: ______)

---

8. Have you spent most of your life in a rural or urban area?

◯ Urban
◯ Rural
◯ Suburban
◯ Other (please describe): ______

---

9. What is your gender?

◯ Male
◯ Female
◯ Non-binary/third gender
◯ Prefer not to say
◯ Prefer to self-describe in a text box ______

10. What is your sexual orientation?

◯ Heterosexual (Straight)
◯ Homosexual (Gay/Lesbian)
◯ Bisexual
◯ Pansexual
◯ Asexual
◯ Queer
◯ Questioning
◯ Prefer not to say
◯ Other (please describe): ______

What is 2 + 2?

◯ 2
◯ 4
◯ 8

---

11. Which of the following best describes where you live?

◯ Apartment
◯ House without backyard
◯ House with backyard
◯ Small property (less than 1,000m^2)
◯ Medium property (1,000m^2-1,0000m^2)
◯ Large property (more than 1,0000m^2))
◯ Other (please describe): ______

---

12. What is your highest level of education?

◯ Primary school
◯ Lower secondary/middle school
◯ Higher secondary/year 12 or equivalent
◯ Vocational qualification
◯ Advanced Diploma
◯ Undergraduate diploma
◯ Bachelor degree
◯ Postgraduate degree (Master's, doctorate)

---

13. What is your main work status?

◯ Student
◯ Employed
◯ Unemployed
◯ Retired
◯ Homemaker/Housewife

---

14. In terms of personal financial wealth, how do you consider yourself?

◯ Well below average
◯ Below average
◯ Average
◯ Above average
◯ Well above average
◯ Prefer not to say

15. Which of the following best describes your belief system?

◯ Atheist
◯ I do not follow an organised religion, but adhere to folk beliefs (e.g., ancestor worship, Mother Goddess worship, etc.)
◯ Buddhist
◯ Catholic
◯ Hoa Hao
◯ Cao Dai
◯ Protestant
◯ Muslim
◯ Other (please describe): ______

---

16. How many dogs do you currently own that live with you?

◯ 0
◯ 1
◯ 2
◯ 3
◯ 4
◯ 5
◯ 6
◯ 7
◯ 8
◯ 9
◯ 10
◯ 11
◯ 12
◯ 13
◯ 14
◯ 15
◯ 16
◯ 17
◯ 18
◯ 19
◯ 20 or more

---

17. How many dogs have previously lived with you over your lifetime?

◯ 0
◯ 1
◯ 2
◯ 3
◯ 4
◯ 5
◯ 6
◯ 7
◯ 8
◯ 9
◯ 10
◯ 11
◯ 12
◯ 13
◯ 14
◯ 15
◯ 16
◯ 17
◯ 18
◯ 19
◯ 20 or more

IDEAL DOG SCALE

Below are statements about the importance of various dog characteristics. Please answer the questions in relation to your ideal dog - an imaginary dog that would be perfect for you.

---

1. What would the sex of your ideal dog be?

◯ Male
◯ Female
◯ The sex of the dog is not important

---

2. What would the sexual status of your ideal dog be?

◯ De-sexed (neutered)
◯ Not de-sexed
◯ Desexing status is not important

---

3. What is your preference regarding the length of your ideal dog's hair/fur (tick all that apply)?

☐ Hairless
☐ Short
☐ Medium
☐ Long
☐ Coat length is not important

---

4. What is your preference regarding the texture of your ideal dog's hair/fur (tick all that apply)?

☐ Smooth hair/fur
☐ Wavey hair/fur
☐ Curly hair/fur
☐ Corded hair/fur
☐ Hair/fur texture is not important

---

5. What is your preference regarding how much your ideal dog sheds its hair/fur?

◯ No shedding
◯ Low shedding
◯ Moderate shedding
◯ Heavy shedding
◯ The shedding amount is not important

---

6. What coat colour would your ideal dog have?

◯ Black
◯ White
◯ Brown
◯ Multi-coloured
◯ Other (please specify): ______
◯ Colour is not important

---

7. What size would your ideal dog be?

◯ Tiny (0-3kg)
◯ Small (4-10kg)
◯ Medium (10-20kg)
◯ Large (20-40kg)
◯ X-large (40+kg)
◯ Size is not important

8. What would the breed type of your ideal dog be?

◯ Purebred
◯ Mixed breed
◯ Designer dog
◯ Breed type is not important

9. What age would you acquire your ideal dog?

◯ Acquired as a puppy
◯ Acquired as an adult
◯ Age of acquisition is not important

10. To maintain your ideal dog, how much would it cost per week?

◯ $0-10
◯ $11-20
◯ $21-30
◯ $31+
◯ Cost is not important

11. How many minutes of exercise would your ideal dog require per day?

◯ 0
◯ 1-15
◯ 16-30
◯ 31-60
◯ 61+

12. How many minutes of grooming would your ideal dog require per week?

◯ 0
◯ 1-15
◯ 16-30
◯ 31-60
◯ 61+

Please provide your opinion on each item, with 1 being extremely unimportant and 5 being extremely important.

My ideal dog...

	Extremely unimportant	Unimportant	Neither important nor unimportant	Important	Extremely important
1. Is fully housetrained (never soils in the house)	◯	◯	◯	◯	◯
2. Does not bark inappropriately	◯	◯	◯	◯	◯
3. Never jumps on people	◯	◯	◯	◯	◯
4. Comes to me when they are called	◯	◯	◯	◯	◯

5. Is friendly towards strangers	○	○	○	○	○
6. Walks calmly without pulling on the leash	○	○	○	○	○
7. Let's me groom them easily	○	○	○	○	○
8. Allows the vet to examine them	○	○	○	○	○
9. Travels calmly and quietly in motorcycle or other vehicles	○	○	○	○	○
10. Is not destructive when left alone	○	○	○	○	○
11. Barks at people who enter my property	○	○	○	○	○
12. Is confident in new surroundings	○	○	○	○	○
13. Will bite people on command	○	○	○	○	○
14. Remains calm during thunderstorms or fireworks	○	○	○	○	○
15. Does not eat their own faeces	○	○	○	○	○
16. Does not eat other animals faeces	○	○	○	○	○
17. Does not scavenge things found in the street	○	○	○	○	○
18. Does not bark at strangers in public areas	○	○	○	○	○
19. Does not growl at strangers in public areas	○	○	○	○	○
20. Learns new tasks quickly	○	○	○	○	○
21. Does not chase wildlife or farm animals	○	○	○	○	○
22. Has hunting capabilities	○	○	○	○	○
23. Has high energy levels	○	○	○	○	○
24. Is safe with children	○	○	○	○	○
25. Behaves calmly most of the time	○	○	○	○	○
26. Does not exhibit inappropriate sexual behaviours	○	○	○	○	○
27. Is not overly excitable	○	○	○	○	○
28. Does not dig inappropriately	○	○	○	○	○
29. Does not beg for food	○	○	○	○	○
30. Shows affection toward me	○	○	○	○	○
31. Lives until they are at least 10 years old	○	○	○	○	○
32. Is physically healthy	○	○	○	○	○

33. Enjoys being petted	○	○	○	○	○
34. Is friendly with other dogs	○	○	○	○	○
35. Is protective of myself and my family	○	○	○	○	○
36. Enjoys large amounts of exercise	○	○	○	○	○
37. Enjoys obedience training	○	○	○	○	○
38. Does not escape from my property	○	○	○	○	○
39. Is physically impressive to look at	○	○	○	○	○
40. Does not fight with other dog s	○	○	○	○	○
41. Enjoys being cuddled and hugged	○	○	○	○	○
42. Likes to play rough and tumble games	○	○	○	○	○
43. Tolerates being left alone for long periods	○	○	○	○	○
44. Is constantly attentive to me	○	○	○	○	○

a

DOG DEMOGRAPHIC QUESTIONS

The following questions ask you about your CURRENT dog. If you have more than one dog, please answer the following questions about your favourite dog.

---

1. Does your dog have a name?

○ No
○ Yes (please write): ______

---

2. What is your dog's current age (count by years)?

○ Less than 1 year
○ Between 1 - 2 years
○ Between 2 - 8 years
○ Between 8 - 12 years
○ 12+ years
○ Don't know

---

3. What was your dog's age at acquisition?

○ Less than 1 month
○ Between 1 - 4 months
○ Between 4 - 12 months
○ Between 1 - 3 years
○ Over 3 years
○ Don't know

---

4. What is the sex of your dog?

○ Male
○ Female
○ Don't know

5. Is your dog desexed/castrated?

◯ Yes
◯ No
◯ Don't know

6. Where did your dog come from?

◯ Pet shop
◯ Breeder
◯ Shelter/rescue
◯ Friend/family
◯ Bred myself
◯ Gift
◯ Inherited
◯ Found
◯ Other (please specify): ______

7. Which of the following best describes your dog?

◯ Pet/companion animal
◯ Guard dog
◯ Breeding dog
◯ Working (farm/hunting/ratting) dog
◯ Other (please specify): ______

8. What length is your dog's hair/fur?

◯ Hairless
◯ Short
◯ Medium
◯ Long

9. What is the texture of your dog's hair/fur?

◯ Smooth hair/fur
◯ Wavey hair/fur
◯ Curly hair/fur
◯ Corded hair/fur

10. Relative to other dogs, how much does your dog shed their hair/fur?

◯ No shedding
◯ Low shedding
◯ Moderate shedding
◯ Heavy shedding

11. What is your dog's coat colour?

◯ Black
◯ White
◯ Brown
◯ Multi-coloured
◯ Other (please specify): ______

12. What size is your dog?

◯ Tiny (0-3kg)
◯ Small (4-10kg)
◯ Medium (10-20kg)
◯ Large (20-40kg)
◯ X-large (40+kg)

13. What breed type is your dog?

◯ Purebred
◯ Mixed breed
◯ Designer dog

14. How much does it cost to maintain your dog per week?

◯ $0-10
◯ $11-20
◯ $21-30
◯ $31+

15. How many mintues of exercise does your dog require per day?

◯ 0
◯ 1-15
◯ 16-30
◯ 31-60
◯ 61+

---

16. How many minutes of grooming does your dog require per week?

◯ 0
◯ 1-15
◯ 16-30
◯ 31-60
◯ 61+

---

17. Where does your dog spend most of its time?

◯ On my property (inside the house and/or the yard)
◯ Free roaming
◯ Other (please specify): ______

---

18. When on your property, where does your dog spend most of its time?

◯ Inside my house
◯ Inside my yard
◯ Inside both the house and yard equally
◯ Other (please specify): ______

---

19. How satisfied are you with your dog's behaviour?

◯ Very dissatisfied
◯ Dissatisfied
◯ Neither satisfied nor dissatisfied
◯ Satisfied
◯ Very satisfied

---

20. How satisfied are your with your dog's health?

◯ Very dissatisfied
◯ Dissatisfied
◯ Neither satisfied nor dissatisfied
◯ Satisfied
◯ Very satisfied

---

21. How satisfied are you with your dog's physical appearance?

◯ Very dissatisfied
◯ Dissatisfied
◯ Neither satisfied nor dissatisfied
◯ Satisfied
◯ Very satisfied

22. Relative to other dog owners, which of the following best describes the quality of your relationship with your dog?

- ◯ Much worse
- ◯ Somewhat worse
- ◯ Slightly worse
- ◯ About the same
- ◯ Slightly better
- ◯ Somewhat better
- ◯ Much better
